# Dimethyl­ammonium 5,5-dimethyl-3-oxo-2-(3,3,6,6-tetra­methyl-1,8-dioxo-2,3,4,5,6,7,8,9-octa­hydro-1*H*-xanthen-9-yl)cyclo­hex-1-enolate 9-(2-hydr­oxy-4,4-dimethyl-6-oxocyclo­hex-1-en­yl)-3,3,6,6-tetra­methyl-3,4,5,6,7,9-hexa­hydro-1*H*-xanthene-1,8(2*H*)-dione *n*-hexane hemisolvate monohydrate

**DOI:** 10.1107/S1600536810003107

**Published:** 2010-01-30

**Authors:** Noorhafizah Hasanudin, Aisyah Saad Abdul Rahim, Salizawati Muhamad Salhimi, Jia Hao Goh, Hoong-Kun Fun

**Affiliations:** a School of Pharmaceutical Sciences, Universiti Sains Malaysia, 11800 USM, Penang, Malaysia; b X-ray Crystallography Unit, School of Physics, Universiti Sains Malaysia, 11800 USM, Penang, Malaysia

## Abstract

The main mol­ecule of the title compound, C_2_H_8_N^+^·C_25_H_31_O_5_^−^·C_25_H_32_O_5_·0.5C_6_H_14_·H_2_O, exists as two crystallographically independent mol­ecules, the hydr­oxy group of one being deprotonated. The pyran rings of both independent units adopt boat conformations. One of the two cyclo­hexene rings of the xanthene unit adopts an envelope conformation whereas the other is in a half-chair conformation. The cyclo­hexene ring attached to the xanthene unit adopts an envelope conformation. The *n*-hexane solvent mol­ecule is disordered about a crystallographic glide plane and the symmetry-independent components are again disordered over two positions, each with an occupancy of 0.25. In the crystal structure, the xanthene derivatives are linked by O—H⋯O, N—H⋯O and C—H⋯O hydrogen bonds, forming a three-dimensional network with channels along the *a* axis. The dimethyl­ammonium cations and water mol­ecules lie in small channels and are linked to the framework *via* O—H.·O and N—H⋯O hydrogen bonds. The *n*-hexane solvent mol­ecules occupy large channels.

## Related literature

For general background and the preparation and applications of the title compound, see: Ashry *et al.* (2006 ▶); Rubinov *et al.* (1999 ▶); Saitoh *et al.* (2006 ▶). For ring conformations, see: Cremer & Pople (1975 ▶). For related structures, see: Jeyakanthan *et al.* (1999 ▶); Odabaşoğlu *et al.* (2008 ▶). For the stability of the temperature controller used for the data collection, see: Cosier & Glazer (1986 ▶).
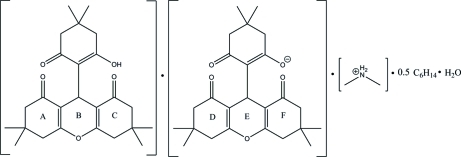


## Experimental

### 

#### Crystal data

C_2_H_8_N^+^·C_25_H_31_O_5_^−^·C_25_H_32_O_5_·0.5C_6_H_14_·H_2_O
*M* *_r_* = 931.20
Orthorhombic, 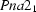

*a* = 11.2438 (2) Å
*b* = 20.1671 (3) Å
*c* = 23.6474 (3) Å
*V* = 5362.16 (14) Å^3^
*Z* = 4
Mo *K*α radiation
μ = 0.08 mm^−1^
*T* = 100 K
0.48 × 0.38 × 0.20 mm

#### Data collection

Bruker SMART APEXII CCD area-detector diffractometer
Absorption correction: multi-scan (*SADABS*; Bruker, 2009 ▶) *T*_min_ = 0.963, *T*_max_ = 0.984
31958 measured reflections
8021 independent reflections
6458 reflections with *I* > 2σ(*I*)
*R*_int_ = 0.040

#### Refinement

*R*[*F* ^2^ > 2σ(*F* ^2^)] = 0.052
*wR*(*F* ^2^) = 0.145
*S* = 1.03
8021 reflections
640 parameters
19 restraints
H-atom parameters constrained
Δρ_max_ = 0.48 e Å^−3^
Δρ_min_ = −0.28 e Å^−3^

### 

Data collection: *APEX2* (Bruker, 2009 ▶); cell refinement: *SAINT* (Bruker, 2009 ▶); data reduction: *SAINT*; program(s) used to solve structure: *SHELXTL* (Sheldrick, 2008 ▶); program(s) used to refine structure: *SHELXTL*; molecular graphics: *SHELXTL*; software used to prepare material for publication: *SHELXTL* and *PLATON* (Spek, 2009 ▶).

## Supplementary Material

Crystal structure: contains datablocks global, I. DOI: 10.1107/S1600536810003107/ci5021sup1.cif

Structure factors: contains datablocks I. DOI: 10.1107/S1600536810003107/ci5021Isup2.hkl

Additional supplementary materials:  crystallographic information; 3D view; checkCIF report

## Figures and Tables

**Table 1:** Hydrogen-bond geometry (Å, °)

*D*—H⋯*A*	*D*—H	H⋯*A*	*D*⋯*A*	*D*—H⋯*A*
O5*B*—H5*OB*⋯O5*A*	0.82	1.69	2.488 (2)	163
O1*W*—H2*W*1⋯O4*A*^i^	0.89	2.03	2.918 (3)	175
N1—H1*N*1⋯O4*A*^ii^	0.88	1.94	2.755 (3)	155
N1—H2*N*1⋯O1*W*	0.87	2.01	2.860 (3)	167
C4*B*—H4*C*⋯O2*A*^i^	0.97	2.48	3.353 (4)	150
C12*A*—H12*B*⋯O3*A*^iii^	0.97	2.41	3.261 (3)	146
C12*B*—H12*C*⋯O3*B*^iv^	0.97	2.36	3.238 (3)	150
C18*A*—H18*A*⋯O5*B*	0.97	2.55	3.227 (3)	126
C26—H26*C*⋯O4*B*	0.96	2.42	3.244 (4)	144
C27—H27*B*⋯O3*B*	0.96	2.34	3.055 (4)	131

Symmetry codes: (i) 
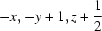
; (ii) 
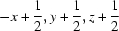
; (iii) 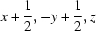; (iv) 
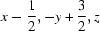
.

**Fig. 1.**
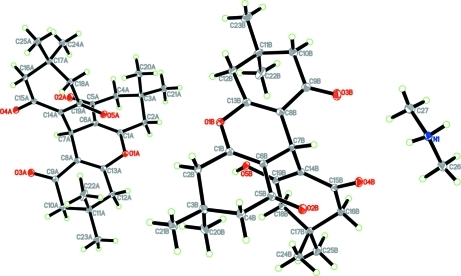
The asymmetric unit of the title compound, showing the atomic numbering scheme. Displacement ellipsoids are drawn at the 30% probability level. For clarity, n-hexane and water molecule are not shown.

**Fig. 2.**
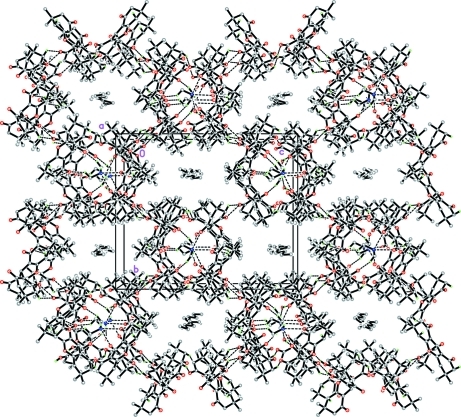
The crystal structure of the title compound, viewed along the a axis, showing a three-dimensional extended framework. Only the major component of the n-hexane solvent is shown. H atoms not involved in intermolecular interactions (dashed lines) have been omitted for clarity.

## supplementary crystallographic information

### Comment

Dimedone derivatives are useful to determine airbone aldehydes (Saitoh *et al.*, 2006) and these compounds are useful precursors of antibiotics and anticancer agents (Rubinov *et al.*, 1999). Dimedone can also be further elaborated by condensing with 2-formyldimedone or triethyl orthoformate to form xanthene derivatives (Ashry *et al.*, 2006). As part of an ongoing study on such compounds, in this paper, we present the crystal structure of the title compound.

The asymmetric unit of the title compound (Fig. 1) comprises of two crystallographically independent 9-(2-hydroxy-4,4-dimethyl-6-oxocyclohex-1-enyl)-3,3,6,6-tetramethyl-3,4,5,6,7,9-hexahydro-1*H*-xanthene-1,8(2*H*)-dione molecules, a dimethylammonia molecule, a partially occupied *n*-hexane solvent molecule and a water molecule. A proton is transferred from the atom O5A of hydroxy group in molecule *A* to the dimethylammonia molecule resulted in the formation of ions. The *n*-hexane solvent molecule is disordered over two positions each with an occupancy of 0.25. Both of these disordered components are further disordered over a crystallographic glide plane [symmetry code: x-1/2, 3/2-y, z]. Each main molecule contains a fused three-ring xanthene moiety and a cyclohexene moiety attached to the xanthene moiety. In the xanthene moiety, the cyclohexene ring A (C1-C6), the 4*H*-pyran ring B (O1/C1/C6-C8/C13) and the cyclohexene ring C (C8-C13) are not planar, having total puckering amplitudes Q of 0.495 (3), 0.238 (2) and 0.474 (3) Å, respectively; the corresponding values for rings D, E and F in molecule *B* are 0.482 (3), 0.196 (2) and 0.478 (3), respectively. Rings A, B and C adopt envelope [θ = 121.4 (3)° and φ = 307.8 (4)°], boat [θ = 73.3 (5)° and φ = 180.0 (1)°] and half-chair [θ = 55.6 (4)° and φ = 161.4 (4)°] conformations, respectively. Rings D, E and F in molecule *B* adopt similar conformations; the θ/φ values of 53.9 (4)°/127.5 (4)°, 108.2 (6)°/0.6 (8)° and 122.9 (4)°/340.7 (4)°, respectively. The cyclohexene ring (C14-C19) attached to the xanthene moiety is in an envelope conformation with puckering parameters Q = 0.466 (3) Å, θ = 59.6 (4)° and φ = 189.8 (4)° for molecule *A* and Q = 0.482 (3) Å, θ = 126.9 (4)° and φ = 353.3 (4)° for molecule *B*. The bond lengths and angles are comparable to those observed in related structures (Jeyakanthan *et al.*, 1999; Odabaşoğlu *et al.*, 2008).

In the crystal structure (Fig. 2), the *n*-hexane solvent molecule is not involved in intermolecular hydrogen bonding. All the other molecules and ions are linked by intermolecular O5B—H5OB···O5A, O1W—H2W1···O4A, N1—H2N1···O1W, C4B—H4C···O2A, C12A—H12B···O3A, C12B—H12C···O3B, C18A—H18A···O5B, C26—H26C···O4B and C27—H27B···O3B hydrogen bonds (Table 1) into three-dimensional supramolecular network such that *n*-hexane molecules are surrounded by these ions and molecules.

### Experimental

The title compound (Ashry *et al.*, 2006) was obtained as a side product by refluxing a solution of dimethylformamide dimethylacetal (10 ml, 73.0 mmol) with 5,5-dimethylcyclohexane-1,3-dione (10.0 g, 71.4 mmol) in 1,2-dimethoxyethane (70 ml) for 2 h. The solution was evaporated *in vacuo* to yield a crude material. Good quality single crystals were obtained by recrystallization from EtOAc/Hexane (1:1).

### Refinement

The *n*-hexane solvent molecule is disordered over two positions each with an occupancy of 0.25; all atoms refined isotropically. The 1-2 and 1-3 C—C distances in the disordered components were restrained to 1.52 (1) and 2.50 (1) Å, respectively. H atoms bound to O1W and N1 were located in a difference Fourier map and then constrained to ride with the parent atom with *U*_iso_(H) = 1.5*U*_eq_(O) and 1.2 *U*_eq_(N). The remaining H atoms were placed in the calculated positions (C—H = 0.96–0.98 Å) and refined using a riding model with *U*_iso_(H) = 1.2 or 1.5 *U*_eq_(C). A rotating group model was used for the hydroxy and non-disordered methyl groups. Reflections 011 and 020 were omitted as their intensities were affected by the beam backstop. In the absence of significant anomalous dispersion, 4774 Friedel pairs were merged for the final refinement.

### Crystal data

**Table:**

C_2_H_8_N^+^·C_25_H_31_O_5_^−^·C_25_H_32_O_5_·0.5C_6_H_14_·H_2_O	*F*(000) = 2020
*M**_r_* = 931.20	*D*_x_ = 1.153 Mg m^−^^3^
Orthorhombic, *P**n**a*2_1_	Mo *K*α radiation, λ = 0.71073 Å
Hall symbol: P 2c -2n	Cell parameters from 8018 reflections
*a* = 11.2438 (2) Å	θ = 2.3–27.8°
*b* = 20.1671 (3) Å	µ = 0.08 mm^−^^1^
*c* = 23.6474 (3) Å	*T* = 100 K
*V* = 5362.16 (14) Å^3^	Block, green
*Z* = 4	0.48 × 0.38 × 0.20 mm

### Data collection

**Table:**

Bruker SMART APEXII CCD area-detector diffractometer	8021 independent reflections
Radiation source: fine-focus sealed tube	6458 reflections with *I* > 2σ(*I*)
graphite	*R*_int_ = 0.040
φ and ω scans	θ_max_ = 30.1°, θ_min_ = 2.1°
Absorption correction: multi-scan (*SADABS*; Bruker, 2009)	*h* = −15→15
*T*_min_ = 0.963, *T*_max_ = 0.984	*k* = −24→28
31958 measured reflections	*l* = −33→23

### Refinement

**Table:**

Refinement on *F*^2^	Primary atom site location: structure-invariant direct methods
Least-squares matrix: full	Secondary atom site location: difference Fourier map
*R*[*F*^2^ > 2σ(*F*^2^)] = 0.052	Hydrogen site location: inferred from neighbouring sites
*wR*(*F*^2^) = 0.145	H-atom parameters constrained
*S* = 1.03	*w* = 1/[σ^2^(*F*_o_^2^) + (0.0895*P*)^2^ + 0.2048*P*] where *P* = (*F*_o_^2^ + 2*F*_c_^2^)/3
8021 reflections	(Δ/σ)_max_ = 0.001
640 parameters	Δρ_max_ = 0.48 e Å^−^^3^
19 restraints	Δρ_min_ = −0.28 e Å^−^^3^

### Special details

**Table:**

Experimental. The crystal was placed in the cold stream of an Oxford Cyrosystems Cobra open-flow nitrogen cryostat (Cosier & Glazer, 1986) operating at 100.0 (1)K.
Geometry. All esds (except the esd in the dihedral angle between two l.s. planes) are estimated using the full covariance matrix. The cell esds are taken into account individually in the estimation of esds in distances, angles and torsion angles; correlations between esds in cell parameters are only used when they are defined by crystal symmetry. An approximate (isotropic) treatment of cell esds is used for estimating esds involving l.s. planes.
Refinement. Refinement of F^2^ against ALL reflections. The weighted R-factor wR and goodness of fit S are based on F^2^, conventional R-factors R are based on F, with F set to zero for negative F^2^. The threshold expression of F^2^ > 2sigma(F^2^) is used only for calculating R-factors(gt) etc. and is not relevant to the choice of reflections for refinement. R-factors based on F^2^ are statistically about twice as large as those based on F, and R- factors based on ALL data will be even larger.

### Fractional atomic coordinates and isotropic or equivalent isotropic displacement parameters (Å^2^)

**Table:**

	*x*	*y*	*z*	*U*_iso_*/*U*_eq_	Occ. (<1)
O1A	0.40532 (16)	0.38960 (9)	0.07400 (7)	0.0230 (4)	
O2A	0.20587 (19)	0.44391 (10)	−0.09549 (8)	0.0320 (5)	
O3A	0.07499 (18)	0.25143 (9)	0.03492 (8)	0.0279 (4)	
O4A	−0.05916 (17)	0.36915 (9)	−0.03060 (8)	0.0264 (4)	
O5A	0.19677 (17)	0.46088 (9)	0.10553 (7)	0.0239 (4)	
C1A	0.3864 (2)	0.42668 (12)	0.02593 (11)	0.0219 (5)	
C2A	0.4877 (2)	0.47346 (13)	0.01506 (11)	0.0254 (5)	
H2A	0.5539	0.4491	−0.0011	0.030*	
H2B	0.5139	0.4925	0.0506	0.030*	
C3A	0.4517 (3)	0.52930 (13)	−0.02534 (11)	0.0254 (5)	
C4A	0.3920 (3)	0.49699 (14)	−0.07642 (11)	0.0266 (5)	
H4A	0.3657	0.5316	−0.1020	0.032*	
H4B	0.4505	0.4703	−0.0963	0.032*	
C5A	0.2873 (2)	0.45387 (12)	−0.06170 (11)	0.0232 (5)	
C6A	0.2893 (2)	0.41933 (12)	−0.00681 (10)	0.0200 (5)	
C7A	0.1862 (2)	0.37597 (12)	0.00998 (10)	0.0194 (5)	
H7A	0.1596	0.3519	−0.0237	0.023*	
C8A	0.2320 (2)	0.32568 (12)	0.05230 (10)	0.0211 (5)	
C9A	0.1683 (2)	0.26346 (12)	0.05998 (11)	0.0218 (5)	
C10A	0.2249 (3)	0.21248 (13)	0.09860 (12)	0.0296 (6)	
H10A	0.2829	0.1872	0.0772	0.035*	
H10B	0.1640	0.1819	0.1115	0.035*	
C11A	0.2865 (3)	0.24320 (14)	0.15039 (12)	0.0286 (6)	
C12A	0.3784 (2)	0.29328 (13)	0.12886 (11)	0.0255 (5)	
H12A	0.4041	0.3209	0.1602	0.031*	
H12B	0.4474	0.2694	0.1149	0.031*	
C13A	0.3317 (2)	0.33670 (12)	0.08282 (11)	0.0217 (5)	
C14A	0.0810 (2)	0.41625 (12)	0.03236 (11)	0.0202 (5)	
C15A	−0.0335 (2)	0.41081 (11)	0.00663 (10)	0.0203 (5)	
C16A	−0.1331 (2)	0.45688 (13)	0.02524 (12)	0.0251 (5)	
H16A	−0.1869	0.4320	0.0493	0.030*	
H16B	−0.1774	0.4703	−0.0080	0.030*	
C17A	−0.0947 (2)	0.51939 (12)	0.05702 (11)	0.0217 (5)	
C18A	−0.0049 (2)	0.49856 (13)	0.10225 (11)	0.0242 (5)	
H18A	0.0272	0.5381	0.1200	0.029*	
H18B	−0.0460	0.4733	0.1312	0.029*	
C19A	0.0967 (2)	0.45751 (12)	0.07962 (10)	0.0210 (5)	
C20A	0.3661 (3)	0.57760 (14)	0.00350 (13)	0.0329 (6)	
H20A	0.3420	0.6110	−0.0231	0.049*	
H20B	0.4053	0.5982	0.0350	0.049*	
H20C	0.2975	0.5539	0.0166	0.049*	
C21A	0.5624 (3)	0.56702 (16)	−0.04494 (13)	0.0345 (7)	
H21A	0.5401	0.5992	−0.0729	0.052*	
H21B	0.6183	0.5364	−0.0610	0.052*	
H21C	0.5980	0.5891	−0.0132	0.052*	
C22A	0.1961 (3)	0.2771 (2)	0.18799 (13)	0.0423 (8)	
H22A	0.1609	0.3136	0.1680	0.063*	
H22B	0.2347	0.2933	0.2215	0.063*	
H22C	0.1352	0.2460	0.1984	0.063*	
C23A	0.3510 (3)	0.18878 (17)	0.18375 (16)	0.0439 (8)	
H23A	0.2936	0.1590	0.1999	0.066*	
H23B	0.3972	0.2086	0.2134	0.066*	
H23C	0.4026	0.1646	0.1588	0.066*	
C24A	−0.0372 (3)	0.56907 (14)	0.01634 (12)	0.0315 (6)	
H24A	0.0290	0.5485	−0.0023	0.047*	
H24B	−0.0100	0.6070	0.0372	0.047*	
H24C	−0.0946	0.5829	−0.0113	0.047*	
C25A	−0.2012 (3)	0.55234 (16)	0.08483 (13)	0.0333 (6)	
H25A	−0.2406	0.5210	0.1089	0.050*	
H25B	−0.2554	0.5674	0.0562	0.050*	
H25C	−0.1748	0.5895	0.1069	0.050*	
O1B	−0.00738 (16)	0.60448 (8)	0.22261 (7)	0.0212 (4)	
O2B	0.23071 (18)	0.53361 (9)	0.37642 (7)	0.0266 (4)	
O3B	0.3163 (2)	0.74774 (11)	0.25737 (9)	0.0367 (5)	
O4B	0.44844 (17)	0.61828 (9)	0.33352 (8)	0.0272 (4)	
O5B	0.21200 (16)	0.54438 (9)	0.18264 (7)	0.0230 (4)	
H5OB	0.2192	0.5202	0.1549	0.035*	
C1B	0.0201 (2)	0.56519 (12)	0.26823 (10)	0.0200 (5)	
C2B	−0.0728 (2)	0.51373 (12)	0.27835 (11)	0.0229 (5)	
H2C	−0.1048	0.4992	0.2423	0.027*	
H2D	−0.1373	0.5330	0.3000	0.027*	
C3B	−0.0228 (2)	0.45368 (12)	0.31038 (11)	0.0242 (5)	
C4B	0.0443 (3)	0.48012 (13)	0.36252 (11)	0.0254 (5)	
H4C	−0.0123	0.5009	0.3879	0.031*	
H4D	0.0801	0.4431	0.3824	0.031*	
C5B	0.1397 (2)	0.52953 (12)	0.34798 (10)	0.0212 (5)	
C6B	0.1197 (2)	0.57298 (12)	0.29920 (10)	0.0193 (5)	
C7B	0.2158 (2)	0.62140 (11)	0.28249 (10)	0.0180 (4)	
H7B	0.2412	0.6453	0.3165	0.022*	
C8B	0.1635 (2)	0.67112 (12)	0.24145 (11)	0.0202 (5)	
C9B	0.2234 (3)	0.73471 (13)	0.23309 (12)	0.0260 (5)	
C10B	0.1621 (3)	0.78421 (14)	0.19539 (14)	0.0331 (6)	
H10C	0.2203	0.8161	0.1821	0.040*	
H10D	0.1033	0.8081	0.2174	0.040*	
C11B	0.1011 (3)	0.75288 (15)	0.14464 (14)	0.0326 (6)	
C12B	0.0122 (2)	0.70132 (13)	0.16690 (12)	0.0276 (6)	
H12C	−0.0578	0.7241	0.1811	0.033*	
H12D	−0.0127	0.6730	0.1359	0.033*	
C13B	0.0629 (2)	0.65929 (11)	0.21284 (11)	0.0209 (5)	
C14B	0.3234 (2)	0.58432 (12)	0.25854 (10)	0.0188 (5)	
C15B	0.4346 (2)	0.58579 (12)	0.28963 (11)	0.0212 (5)	
C16B	0.5389 (2)	0.54591 (12)	0.26644 (11)	0.0233 (5)	
H16C	0.5916	0.5344	0.2974	0.028*	
H16D	0.5833	0.5734	0.2402	0.028*	
C17B	0.5006 (2)	0.48219 (12)	0.23613 (11)	0.0234 (5)	
C18B	0.4128 (2)	0.50197 (13)	0.18981 (11)	0.0245 (5)	
H18C	0.4553	0.5256	0.1603	0.029*	
H18D	0.3793	0.4622	0.1732	0.029*	
C19B	0.3128 (2)	0.54520 (12)	0.21138 (10)	0.0194 (5)	
C20B	0.0617 (3)	0.41407 (14)	0.27239 (13)	0.0301 (6)	
H20D	0.0192	0.3983	0.2399	0.045*	
H20E	0.0929	0.3770	0.2931	0.045*	
H20F	0.1260	0.4421	0.2604	0.045*	
C21B	−0.1251 (3)	0.40906 (14)	0.33016 (13)	0.0322 (6)	
H21D	−0.1675	0.3925	0.2979	0.048*	
H21E	−0.1782	0.4342	0.3536	0.048*	
H21F	−0.0935	0.3725	0.3514	0.048*	
C22B	0.1928 (3)	0.7193 (2)	0.10600 (13)	0.0420 (8)	
H22D	0.2349	0.6859	0.1269	0.063*	
H22E	0.2482	0.7518	0.0924	0.063*	
H22F	0.1528	0.6991	0.0745	0.063*	
C23B	0.0340 (3)	0.80580 (18)	0.11072 (19)	0.0509 (10)	
H23D	0.0892	0.8385	0.0972	0.076*	
H23E	−0.0240	0.8267	0.1346	0.076*	
H23F	−0.0052	0.7853	0.0792	0.076*	
C24B	0.4438 (3)	0.43408 (13)	0.27822 (13)	0.0297 (6)	
H24D	0.4215	0.3941	0.2590	0.045*	
H24E	0.3744	0.4542	0.2945	0.045*	
H24F	0.4998	0.4238	0.3076	0.045*	
C25B	0.6089 (3)	0.44849 (16)	0.20926 (14)	0.0343 (6)	
H25D	0.5837	0.4094	0.1894	0.051*	
H25E	0.6645	0.4365	0.2383	0.051*	
H25F	0.6462	0.4785	0.1832	0.051*	
O1W	0.30424 (18)	0.65488 (10)	0.43443 (8)	0.0309 (4)	
H1W1	0.3303	0.6161	0.4410	0.046*	
H2W1	0.2280	0.6482	0.4431	0.046*	
N1	0.4847 (2)	0.75319 (10)	0.41903 (9)	0.0224 (4)	
H1N1	0.4923	0.7866	0.4424	0.027*	
H2N1	0.4223	0.7286	0.4234	0.027*	
C26	0.5972 (3)	0.71579 (16)	0.41662 (17)	0.0443 (8)	
H26A	0.6188	0.7016	0.4540	0.066*	
H26B	0.6588	0.7437	0.4016	0.066*	
H26C	0.5874	0.6777	0.3927	0.066*	
C27	0.4533 (3)	0.78416 (15)	0.36451 (12)	0.0357 (7)	
H27A	0.3823	0.8101	0.3690	0.054*	
H27B	0.4399	0.7502	0.3367	0.054*	
H27C	0.5172	0.8123	0.3523	0.054*	
C28	−0.1459 (18)	0.7689 (14)	0.8581 (10)	0.095 (7)*	0.25
H28A	−0.2233	0.7554	0.8708	0.143*	0.25
H28B	−0.1442	0.8162	0.8538	0.143*	0.25
H28C	−0.1289	0.7483	0.8224	0.143*	0.25
C29	−0.0513 (16)	0.7452 (12)	0.8987 (9)	0.091 (7)*	0.25
H29A	−0.0567	0.6980	0.9034	0.110*	0.25
H29B	−0.0719	0.7650	0.9343	0.110*	0.25
C30	0.0716 (16)	0.7662 (11)	0.8792 (8)	0.075 (5)*	0.25
H30A	0.0705	0.8124	0.8693	0.090*	0.25
H30B	0.0896	0.7429	0.8448	0.090*	0.25
C31	0.1606 (17)	0.7533 (15)	0.9274 (9)	0.105 (8)*	0.25
H31A	0.1600	0.7118	0.9473	0.126*	0.25
H31B	0.1689	0.7889	0.9540	0.126*	0.25
C32	0.2838 (17)	0.773 (2)	0.9061 (12)	0.120 (10)*	0.25
H32A	0.2741	0.8105	0.8815	0.144*	0.25
H32B	0.2839	0.7332	0.8839	0.144*	0.25
C33	0.375 (2)	0.768 (4)	0.9532 (17)	0.28 (4)*	0.25
H33A	0.4423	0.7695	0.9283	0.420*	0.25
H33B	0.3720	0.8077	0.9754	0.420*	0.25
H33C	0.3818	0.7302	0.9778	0.420*	0.25
C28X	−0.1944 (19)	0.743 (2)	0.8716 (16)	0.164 (18)*	0.25
H28D	−0.2389	0.7398	0.8371	0.246*	0.25
H28E	−0.2107	0.7055	0.8950	0.246*	0.25
H28F	−0.2169	0.7830	0.8912	0.246*	0.25
C29X	−0.0707 (19)	0.7670 (16)	0.8539 (10)	0.116 (10)*	0.25
H29C	−0.0644	0.8033	0.8279	0.139*	0.25
H29D	−0.0583	0.7279	0.8316	0.139*	0.25
C30X	0.0192 (17)	0.7572 (13)	0.9005 (8)	0.088 (7)*	0.25
H30C	0.0166	0.7177	0.9232	0.105*	0.25
H30D	0.0014	0.7926	0.9263	0.105*	0.25
C31X	0.1456 (17)	0.7710 (14)	0.8791 (8)	0.098 (8)*	0.25
H31C	0.1619	0.8151	0.8662	0.117*	0.25
H31D	0.1731	0.7401	0.8511	0.117*	0.25
C32X	0.2331 (18)	0.760 (2)	0.9278 (9)	0.126 (11)*	0.25
H32C	0.2180	0.7157	0.9414	0.151*	0.25
H32D	0.2094	0.7913	0.9559	0.151*	0.25
C33X	0.3598 (18)	0.7691 (15)	0.9074 (11)	0.101 (8)*	0.25
H33D	0.4098	0.7623	0.9399	0.151*	0.25
H33E	0.3801	0.7375	0.8786	0.151*	0.25
H33F	0.3714	0.8132	0.8932	0.151*	0.25

### Atomic displacement parameters (Å^2^)

**Table:**

	*U*^11^	*U*^22^	*U*^33^	*U*^12^	*U*^13^	*U*^23^
O1A	0.0221 (9)	0.0246 (8)	0.0224 (9)	0.0006 (7)	−0.0049 (7)	0.0015 (7)
O2A	0.0353 (12)	0.0373 (10)	0.0235 (9)	−0.0043 (9)	−0.0110 (8)	0.0036 (8)
O3A	0.0297 (10)	0.0314 (9)	0.0226 (9)	−0.0045 (8)	−0.0035 (8)	0.0001 (8)
O4A	0.0254 (9)	0.0249 (8)	0.0289 (9)	0.0029 (8)	−0.0095 (8)	−0.0076 (7)
O5A	0.0224 (9)	0.0292 (9)	0.0202 (8)	0.0056 (7)	−0.0059 (7)	−0.0081 (7)
C1A	0.0241 (13)	0.0225 (11)	0.0192 (11)	0.0032 (10)	−0.0002 (10)	−0.0007 (9)
C2A	0.0228 (13)	0.0299 (12)	0.0235 (12)	−0.0028 (10)	−0.0046 (10)	0.0009 (10)
C3A	0.0258 (13)	0.0269 (11)	0.0234 (12)	−0.0040 (11)	−0.0018 (10)	0.0022 (10)
C4A	0.0295 (14)	0.0301 (12)	0.0203 (12)	−0.0029 (11)	−0.0023 (10)	0.0036 (10)
C5A	0.0256 (13)	0.0248 (11)	0.0192 (11)	0.0015 (10)	−0.0013 (10)	−0.0012 (9)
C6A	0.0208 (12)	0.0208 (11)	0.0184 (11)	0.0019 (9)	−0.0009 (9)	−0.0021 (9)
C7A	0.0205 (11)	0.0212 (10)	0.0164 (11)	0.0019 (9)	−0.0010 (9)	−0.0025 (9)
C8A	0.0217 (12)	0.0228 (10)	0.0189 (11)	0.0054 (10)	0.0011 (10)	−0.0015 (9)
C9A	0.0260 (13)	0.0234 (11)	0.0159 (10)	0.0019 (10)	0.0019 (10)	−0.0020 (9)
C10A	0.0299 (14)	0.0232 (11)	0.0357 (15)	0.0008 (11)	−0.0042 (12)	0.0065 (11)
C11A	0.0225 (13)	0.0345 (14)	0.0289 (13)	−0.0012 (11)	−0.0028 (11)	0.0109 (11)
C12A	0.0232 (13)	0.0307 (12)	0.0225 (12)	0.0048 (11)	−0.0003 (10)	0.0044 (10)
C13A	0.0202 (12)	0.0247 (11)	0.0201 (11)	0.0038 (10)	0.0029 (10)	−0.0007 (9)
C14A	0.0203 (12)	0.0213 (10)	0.0191 (11)	0.0029 (9)	−0.0032 (9)	−0.0031 (9)
C15A	0.0221 (12)	0.0188 (10)	0.0200 (11)	0.0026 (9)	−0.0022 (9)	−0.0013 (9)
C16A	0.0208 (12)	0.0271 (12)	0.0273 (13)	0.0033 (10)	−0.0036 (10)	−0.0036 (10)
C17A	0.0209 (12)	0.0246 (11)	0.0195 (11)	0.0067 (10)	−0.0040 (9)	−0.0052 (9)
C18A	0.0256 (13)	0.0286 (12)	0.0184 (11)	0.0063 (10)	−0.0028 (10)	−0.0057 (10)
C19A	0.0236 (12)	0.0212 (11)	0.0182 (11)	0.0020 (10)	−0.0034 (10)	−0.0022 (9)
C20A	0.0355 (16)	0.0269 (12)	0.0362 (15)	−0.0031 (12)	0.0002 (13)	−0.0031 (11)
C21A	0.0313 (15)	0.0378 (14)	0.0344 (15)	−0.0138 (13)	−0.0067 (12)	0.0091 (12)
C22A	0.0280 (15)	0.074 (2)	0.0251 (14)	−0.0032 (15)	0.0013 (12)	0.0021 (15)
C23A	0.0373 (17)	0.0426 (17)	0.052 (2)	−0.0061 (15)	−0.0141 (16)	0.0247 (15)
C24A	0.0419 (16)	0.0257 (12)	0.0267 (14)	0.0034 (12)	−0.0031 (12)	−0.0008 (11)
C25A	0.0268 (14)	0.0407 (15)	0.0323 (15)	0.0123 (12)	−0.0049 (12)	−0.0111 (12)
O1B	0.0205 (9)	0.0234 (8)	0.0196 (8)	0.0017 (7)	−0.0009 (7)	0.0009 (7)
O2B	0.0311 (10)	0.0288 (9)	0.0201 (9)	−0.0009 (8)	−0.0039 (8)	0.0000 (7)
O3B	0.0380 (13)	0.0371 (11)	0.0349 (11)	−0.0134 (10)	−0.0096 (10)	0.0076 (9)
O4B	0.0261 (10)	0.0294 (9)	0.0259 (9)	0.0002 (8)	−0.0055 (8)	−0.0084 (8)
O5B	0.0238 (9)	0.0278 (9)	0.0175 (8)	0.0059 (7)	−0.0060 (7)	−0.0078 (7)
C1B	0.0214 (12)	0.0214 (10)	0.0173 (11)	0.0011 (10)	0.0018 (9)	−0.0033 (9)
C2B	0.0229 (12)	0.0250 (11)	0.0207 (12)	−0.0031 (10)	0.0017 (10)	−0.0026 (9)
C3B	0.0256 (13)	0.0252 (11)	0.0217 (12)	−0.0047 (10)	0.0011 (10)	−0.0030 (10)
C4B	0.0314 (14)	0.0280 (12)	0.0170 (11)	−0.0036 (11)	0.0004 (10)	0.0003 (10)
C5B	0.0263 (13)	0.0226 (11)	0.0148 (11)	0.0004 (10)	0.0013 (10)	−0.0038 (9)
C6B	0.0218 (12)	0.0188 (10)	0.0173 (11)	0.0028 (9)	0.0019 (9)	−0.0034 (8)
C7B	0.0176 (11)	0.0199 (10)	0.0166 (10)	0.0002 (9)	0.0012 (9)	−0.0035 (9)
C8B	0.0213 (12)	0.0196 (10)	0.0196 (11)	0.0023 (9)	0.0035 (9)	−0.0023 (9)
C9B	0.0245 (13)	0.0258 (12)	0.0277 (13)	0.0006 (11)	0.0020 (11)	0.0004 (10)
C10B	0.0286 (14)	0.0249 (12)	0.0458 (17)	−0.0026 (12)	0.0006 (13)	0.0093 (12)
C11B	0.0238 (13)	0.0351 (14)	0.0389 (15)	0.0014 (12)	−0.0019 (12)	0.0164 (13)
C12B	0.0225 (13)	0.0289 (12)	0.0315 (14)	0.0042 (11)	−0.0034 (11)	0.0065 (11)
C13B	0.0199 (12)	0.0207 (10)	0.0221 (12)	0.0022 (10)	0.0032 (9)	−0.0028 (9)
C14B	0.0185 (11)	0.0214 (10)	0.0166 (11)	0.0032 (9)	0.0006 (9)	−0.0015 (9)
C15B	0.0229 (12)	0.0203 (10)	0.0203 (11)	0.0006 (10)	−0.0025 (10)	−0.0003 (9)
C16B	0.0209 (12)	0.0271 (12)	0.0218 (12)	0.0011 (10)	−0.0020 (10)	−0.0035 (10)
C17B	0.0212 (12)	0.0270 (12)	0.0222 (12)	0.0067 (10)	−0.0021 (10)	−0.0043 (10)
C18B	0.0247 (13)	0.0305 (12)	0.0183 (11)	0.0082 (11)	−0.0034 (10)	−0.0079 (10)
C19B	0.0200 (12)	0.0228 (11)	0.0155 (11)	0.0026 (9)	−0.0004 (9)	−0.0018 (9)
C20B	0.0285 (14)	0.0265 (12)	0.0352 (15)	−0.0025 (11)	0.0016 (12)	−0.0066 (11)
C21B	0.0368 (16)	0.0292 (13)	0.0305 (14)	−0.0098 (12)	0.0005 (12)	0.0006 (11)
C22B	0.0281 (15)	0.071 (2)	0.0272 (14)	0.0002 (15)	0.0029 (13)	0.0176 (15)
C23B	0.0319 (17)	0.0468 (18)	0.074 (2)	−0.0052 (15)	−0.0131 (17)	0.0333 (19)
C24B	0.0321 (14)	0.0245 (12)	0.0326 (14)	0.0014 (11)	−0.0053 (12)	−0.0016 (11)
C25B	0.0248 (14)	0.0422 (15)	0.0358 (15)	0.0111 (12)	−0.0024 (12)	−0.0118 (13)
O1W	0.0320 (10)	0.0303 (9)	0.0305 (10)	−0.0071 (8)	0.0062 (9)	−0.0044 (8)
N1	0.0248 (11)	0.0230 (9)	0.0196 (10)	−0.0026 (9)	0.0008 (8)	0.0009 (8)
C26	0.0393 (18)	0.0306 (14)	0.063 (2)	0.0081 (14)	−0.0194 (17)	−0.0175 (15)
C27	0.053 (2)	0.0293 (13)	0.0245 (13)	−0.0020 (14)	−0.0030 (13)	0.0025 (11)

### Geometric parameters (Å, °)

**Table:**

O1A—C13A	1.367 (3)	C10B—C11B	1.520 (5)
O1A—C1A	1.377 (3)	C10B—H10C	0.97
O2A—C5A	1.232 (3)	C10B—H10D	0.97
O3A—C9A	1.229 (3)	C11B—C23B	1.533 (4)
O4A—C15A	1.251 (3)	C11B—C22B	1.535 (5)
O5A—C19A	1.283 (3)	C11B—C12B	1.536 (4)
C1A—C6A	1.346 (4)	C12B—C13B	1.492 (4)
C1A—C2A	1.501 (4)	C12B—H12C	0.97
C2A—C3A	1.531 (4)	C12B—H12D	0.97
C2A—H2A	0.97	C14B—C19B	1.371 (3)
C2A—H2B	0.97	C14B—C15B	1.450 (3)
C3A—C4A	1.528 (4)	C15B—C16B	1.524 (4)
C3A—C20A	1.530 (4)	C16B—C17B	1.533 (4)
C3A—C21A	1.530 (4)	C16B—H16C	0.97
C4A—C5A	1.504 (4)	C16B—H16D	0.97
C4A—H4A	0.97	C17B—C18B	1.528 (4)
C4A—H4B	0.97	C17B—C24B	1.530 (4)
C5A—C6A	1.473 (3)	C17B—C25B	1.532 (4)
C6A—C7A	1.505 (4)	C18B—C19B	1.512 (3)
C7A—C8A	1.515 (3)	C18B—H18C	0.97
C7A—C14A	1.530 (3)	C18B—H18D	0.97
C7A—H7A	0.98	C20B—H20D	0.96
C8A—C13A	1.352 (4)	C20B—H20E	0.96
C8A—C9A	1.456 (4)	C20B—H20F	0.96
C9A—C10A	1.515 (4)	C21B—H21D	0.96
C10A—C11A	1.538 (4)	C21B—H21E	0.96
C10A—H10A	0.97	C21B—H21F	0.96
C10A—H10B	0.97	C22B—H22D	0.96
C11A—C22A	1.514 (4)	C22B—H22E	0.96
C11A—C12A	1.532 (4)	C22B—H22F	0.96
C11A—C23A	1.534 (4)	C23B—H23D	0.96
C12A—C13A	1.493 (4)	C23B—H23E	0.96
C12A—H12A	0.97	C23B—H23F	0.96
C12A—H12B	0.97	C24B—H24D	0.96
C14A—C19A	1.404 (3)	C24B—H24E	0.96
C14A—C15A	1.429 (3)	C24B—H24F	0.96
C15A—C16A	1.520 (4)	C25B—H25D	0.96
C16A—C17A	1.530 (3)	C25B—H25E	0.96
C16A—H16A	0.97	C25B—H25F	0.96
C16A—H16B	0.97	O1W—H1W1	0.85
C17A—C25A	1.519 (4)	O1W—H2W1	0.89
C17A—C18A	1.530 (4)	N1—C26	1.474 (4)
C17A—C24A	1.532 (4)	N1—C27	1.476 (4)
C18A—C19A	1.509 (4)	N1—H1N1	0.88
C18A—H18A	0.97	N1—H2N1	0.86
C18A—H18B	0.97	C26—H26A	0.96
C20A—H20A	0.96	C26—H26B	0.96
C20A—H20B	0.96	C26—H26C	0.96
C20A—H20C	0.96	C27—H27A	0.96
C21A—H21A	0.96	C27—H27B	0.96
C21A—H21B	0.96	C27—H27C	0.96
C21A—H21C	0.96	C28—C29	1.511 (10)
C22A—H22A	0.96	C28—H28A	0.96
C22A—H22B	0.96	C28—H28B	0.96
C22A—H22C	0.96	C28—H28C	0.96
C23A—H23A	0.96	C29—C30	1.518 (10)
C23A—H23B	0.96	C29—H29A	0.96
C23A—H23C	0.96	C29—H29B	0.96
C24A—H24A	0.96	C30—C31	1.539 (10)
C24A—H24B	0.96	C30—H30A	0.96
C24A—H24C	0.96	C30—H30B	0.96
C25A—H25A	0.96	C31—C32	1.526 (10)
C25A—H25B	0.96	C31—H31A	0.96
C25A—H25C	0.96	C31—H31B	0.96
O1B—C1B	1.374 (3)	C32—C33	1.516 (10)
O1B—C13B	1.379 (3)	C32—H32A	0.96
O2B—C5B	1.227 (3)	C32—H32B	0.96
O3B—C9B	1.221 (3)	C33—H33A	0.96
O4B—C15B	1.237 (3)	C33—H33B	0.96
O5B—C19B	1.322 (3)	C33—H33C	0.96
O5B—H5OB	0.82	C28X—C29X	1.529 (10)
C1B—C6B	1.347 (4)	C28X—H28D	0.96
C1B—C2B	1.492 (4)	C28X—H28E	0.96
C2B—C3B	1.535 (4)	C28X—H28F	0.96
C2B—H2C	0.97	C29X—C30X	1.508 (10)
C2B—H2D	0.97	C29X—H29C	0.96
C3B—C20B	1.532 (4)	C29X—H29D	0.96
C3B—C21B	1.533 (4)	C30X—C31X	1.534 (10)
C3B—C4B	1.541 (4)	C30X—H30C	0.96
C4B—C5B	1.504 (4)	C30X—H30D	0.96
C4B—H4C	0.97	C31X—C32X	1.530 (10)
C4B—H4D	0.97	C31X—H31C	0.96
C5B—C6B	1.466 (3)	C31X—H31D	0.96
C6B—C7B	1.509 (3)	C32X—C33X	1.516 (10)
C7B—C8B	1.514 (3)	C32X—H32C	0.96
C7B—C14B	1.532 (3)	C32X—H32D	0.96
C7B—H7B	0.98	C33X—H33D	0.96
C8B—C13B	1.339 (4)	C33X—H33E	0.96
C8B—C9B	1.462 (4)	C33X—H33F	0.96
C9B—C10B	1.506 (4)		
			
C13A—O1A—C1A	117.2 (2)	H10C—C10B—H10D	107.7
C6A—C1A—O1A	122.7 (2)	C10B—C11B—C23B	110.2 (3)
C6A—C1A—C2A	125.9 (2)	C10B—C11B—C22B	110.5 (3)
O1A—C1A—C2A	111.4 (2)	C23B—C11B—C22B	109.0 (3)
C1A—C2A—C3A	111.6 (2)	C10B—C11B—C12B	107.7 (2)
C1A—C2A—H2A	109.3	C23B—C11B—C12B	109.3 (2)
C3A—C2A—H2A	109.3	C22B—C11B—C12B	110.1 (3)
C1A—C2A—H2B	109.3	C13B—C12B—C11B	112.7 (2)
C3A—C2A—H2B	109.3	C13B—C12B—H12C	109.1
H2A—C2A—H2B	108.0	C11B—C12B—H12C	109.1
C4A—C3A—C20A	110.3 (2)	C13B—C12B—H12D	109.1
C4A—C3A—C21A	109.2 (2)	C11B—C12B—H12D	109.1
C20A—C3A—C21A	109.3 (2)	H12C—C12B—H12D	107.8
C4A—C3A—C2A	107.2 (2)	C8B—C13B—O1B	122.9 (2)
C20A—C3A—C2A	110.9 (2)	C8B—C13B—C12B	126.1 (2)
C21A—C3A—C2A	109.9 (2)	O1B—C13B—C12B	111.0 (2)
C5A—C4A—C3A	114.0 (2)	C19B—C14B—C15B	120.0 (2)
C5A—C4A—H4A	108.7	C19B—C14B—C7B	120.8 (2)
C3A—C4A—H4A	108.7	C15B—C14B—C7B	118.9 (2)
C5A—C4A—H4B	108.7	O4B—C15B—C14B	123.0 (2)
C3A—C4A—H4B	108.7	O4B—C15B—C16B	119.0 (2)
H4A—C4A—H4B	107.6	C14B—C15B—C16B	118.0 (2)
O2A—C5A—C6A	120.4 (2)	C15B—C16B—C17B	113.2 (2)
O2A—C5A—C4A	121.7 (2)	C15B—C16B—H16C	108.9
C6A—C5A—C4A	117.7 (2)	C17B—C16B—H16C	108.9
C1A—C6A—C5A	117.8 (2)	C15B—C16B—H16D	108.9
C1A—C6A—C7A	122.4 (2)	C17B—C16B—H16D	108.9
C5A—C6A—C7A	119.7 (2)	H16C—C16B—H16D	107.7
C6A—C7A—C8A	107.6 (2)	C18B—C17B—C24B	111.2 (2)
C6A—C7A—C14A	112.2 (2)	C18B—C17B—C25B	109.4 (2)
C8A—C7A—C14A	112.9 (2)	C24B—C17B—C25B	108.7 (2)
C6A—C7A—H7A	108.0	C18B—C17B—C16B	107.3 (2)
C8A—C7A—H7A	108.0	C24B—C17B—C16B	110.2 (2)
C14A—C7A—H7A	108.0	C25B—C17B—C16B	110.1 (2)
C13A—C8A—C9A	118.9 (2)	C19B—C18B—C17B	112.9 (2)
C13A—C8A—C7A	121.6 (2)	C19B—C18B—H18C	109.0
C9A—C8A—C7A	119.5 (2)	C17B—C18B—H18C	109.0
O3A—C9A—C8A	122.0 (2)	C19B—C18B—H18D	109.0
O3A—C9A—C10A	121.0 (2)	C17B—C18B—H18D	109.0
C8A—C9A—C10A	117.0 (2)	H18C—C18B—H18D	107.8
C9A—C10A—C11A	113.3 (2)	O5B—C19B—C14B	120.0 (2)
C9A—C10A—H10A	108.9	O5B—C19B—C18B	117.2 (2)
C11A—C10A—H10A	108.9	C14B—C19B—C18B	122.8 (2)
C9A—C10A—H10B	108.9	C3B—C20B—H20D	109.5
C11A—C10A—H10B	108.9	C3B—C20B—H20E	109.5
H10A—C10A—H10B	107.7	H20D—C20B—H20E	109.5
C22A—C11A—C12A	110.5 (3)	C3B—C20B—H20F	109.5
C22A—C11A—C23A	109.8 (3)	H20D—C20B—H20F	109.5
C12A—C11A—C23A	108.9 (2)	H20E—C20B—H20F	109.5
C22A—C11A—C10A	110.3 (2)	C3B—C21B—H21D	109.5
C12A—C11A—C10A	107.8 (2)	C3B—C21B—H21E	109.5
C23A—C11A—C10A	109.5 (3)	H21D—C21B—H21E	109.5
C13A—C12A—C11A	113.0 (2)	C3B—C21B—H21F	109.5
C13A—C12A—H12A	109.0	H21D—C21B—H21F	109.5
C11A—C12A—H12A	109.0	H21E—C21B—H21F	109.5
C13A—C12A—H12B	109.0	C11B—C22B—H22D	109.5
C11A—C12A—H12B	109.0	C11B—C22B—H22E	109.5
H12A—C12A—H12B	107.8	H22D—C22B—H22E	109.5
C8A—C13A—O1A	123.3 (2)	C11B—C22B—H22F	109.5
C8A—C13A—C12A	125.8 (2)	H22D—C22B—H22F	109.5
O1A—C13A—C12A	110.8 (2)	H22E—C22B—H22F	109.5
C19A—C14A—C15A	119.8 (2)	C11B—C23B—H23D	109.5
C19A—C14A—C7A	119.5 (2)	C11B—C23B—H23E	109.5
C15A—C14A—C7A	120.6 (2)	H23D—C23B—H23E	109.5
O4A—C15A—C14A	124.0 (2)	C11B—C23B—H23F	109.5
O4A—C15A—C16A	116.4 (2)	H23D—C23B—H23F	109.5
C14A—C15A—C16A	119.6 (2)	H23E—C23B—H23F	109.5
C15A—C16A—C17A	116.0 (2)	C17B—C24B—H24D	109.5
C15A—C16A—H16A	108.3	C17B—C24B—H24E	109.5
C17A—C16A—H16A	108.3	H24D—C24B—H24E	109.5
C15A—C16A—H16B	108.3	C17B—C24B—H24F	109.5
C17A—C16A—H16B	108.3	H24D—C24B—H24F	109.5
H16A—C16A—H16B	107.4	H24E—C24B—H24F	109.5
C25A—C17A—C16A	110.6 (2)	C17B—C25B—H25D	109.5
C25A—C17A—C18A	109.7 (2)	C17B—C25B—H25E	109.5
C16A—C17A—C18A	107.7 (2)	H25D—C25B—H25E	109.5
C25A—C17A—C24A	108.6 (2)	C17B—C25B—H25F	109.5
C16A—C17A—C24A	110.5 (2)	H25D—C25B—H25F	109.5
C18A—C17A—C24A	109.9 (2)	H25E—C25B—H25F	109.5
C19A—C18A—C17A	113.7 (2)	H1W1—O1W—H2W1	98.6
C19A—C18A—H18A	108.8	C26—N1—C27	112.9 (3)
C17A—C18A—H18A	108.8	C26—N1—H1N1	109.5
C19A—C18A—H18B	108.8	C27—N1—H1N1	104.4
C17A—C18A—H18B	108.8	C26—N1—H2N1	114.0
H18A—C18A—H18B	107.7	C27—N1—H2N1	98.8
O5A—C19A—C14A	121.4 (2)	H1N1—N1—H2N1	116.4
O5A—C19A—C18A	117.7 (2)	N1—C26—H26A	109.5
C14A—C19A—C18A	120.8 (2)	N1—C26—H26B	109.5
C3A—C20A—H20A	109.5	H26A—C26—H26B	109.5
C3A—C20A—H20B	109.5	N1—C26—H26C	109.5
H20A—C20A—H20B	109.5	H26A—C26—H26C	109.5
C3A—C20A—H20C	109.5	H26B—C26—H26C	109.5
H20A—C20A—H20C	109.5	N1—C27—H27A	109.5
H20B—C20A—H20C	109.5	N1—C27—H27B	109.5
C3A—C21A—H21A	109.5	H27A—C27—H27B	109.5
C3A—C21A—H21B	109.5	N1—C27—H27C	109.5
H21A—C21A—H21B	109.5	H27A—C27—H27C	109.5
C3A—C21A—H21C	109.5	H27B—C27—H27C	109.5
H21A—C21A—H21C	109.5	C29—C28—H28A	110.4
H21B—C21A—H21C	109.5	C29—C28—H28B	111.6
C11A—C22A—H22A	109.5	H28A—C28—H28B	109.5
C11A—C22A—H22B	109.5	C29—C28—H28C	106.4
H22A—C22A—H22B	109.5	H28A—C28—H28C	109.5
C11A—C22A—H22C	109.5	H28B—C28—H28C	109.5
H22A—C22A—H22C	109.5	C28—C29—C30	111.0 (10)
H22B—C22A—H22C	109.5	C28—C29—H29A	110.0
C11A—C23A—H23A	109.5	C30—C29—H29A	111.6
C11A—C23A—H23B	109.5	C28—C29—H29B	104.9
H23A—C23A—H23B	109.5	C30—C29—H29B	111.7
C11A—C23A—H23C	109.5	H29A—C29—H29B	107.2
H23A—C23A—H23C	109.5	C29—C30—C31	108.7 (9)
H23B—C23A—H23C	109.5	C29—C30—H30A	109.4
C17A—C24A—H24A	109.5	C31—C30—H30A	110.7
C17A—C24A—H24B	109.5	C29—C30—H30B	108.2
H24A—C24A—H24B	109.5	C31—C30—H30B	114.0
C17A—C24A—H24C	109.5	H30A—C30—H30B	105.8
H24A—C24A—H24C	109.5	C32—C31—C30	107.6 (9)
H24B—C24A—H24C	109.5	C32—C31—H31A	113.3
C17A—C25A—H25A	109.5	C30—C31—H31A	120.4
C17A—C25A—H25B	109.5	C32—C31—H31B	86.1
H25A—C25A—H25B	109.5	C30—C31—H31B	114.9
C17A—C25A—H25C	109.5	H31A—C31—H31B	109.4
H25A—C25A—H25C	109.5	C33—C32—C31	110.7 (10)
H25B—C25A—H25C	109.5	C33—C32—H32A	125.1
C1B—O1B—C13B	117.7 (2)	C31—C32—H32A	107.6
C19B—O5B—H5OB	109.5	C33—C32—H32B	110.2
C6B—C1B—O1B	123.1 (2)	C31—C32—H32B	87.9
C6B—C1B—C2B	125.2 (2)	H32A—C32—H32B	109.1
O1B—C1B—C2B	111.7 (2)	C32—C33—H33A	94.7
C1B—C2B—C3B	111.8 (2)	C32—C33—H33B	108.8
C1B—C2B—H2C	109.3	H33A—C33—H33B	109.5
C3B—C2B—H2C	109.3	C32—C33—H33C	123.6
C1B—C2B—H2D	109.3	H33A—C33—H33C	109.5
C3B—C2B—H2D	109.3	H33B—C33—H33C	109.5
H2C—C2B—H2D	107.9	C29X—C28X—H28D	105.3
C20B—C3B—C21B	109.8 (2)	C29X—C28X—H28E	125.5
C20B—C3B—C2B	110.4 (2)	H28D—C28X—H28E	109.5
C21B—C3B—C2B	109.8 (2)	C29X—C28X—H28F	96.4
C20B—C3B—C4B	110.2 (2)	H28D—C28X—H28F	109.5
C21B—C3B—C4B	109.0 (2)	H28E—C28X—H28F	109.5
C2B—C3B—C4B	107.5 (2)	C30X—C29X—C28X	111.7 (10)
C5B—C4B—C3B	113.3 (2)	C30X—C29X—H29C	121.3
C5B—C4B—H4C	108.9	C28X—C29X—H29C	118.8
C3B—C4B—H4C	108.9	C30X—C29X—H29D	101.4
C5B—C4B—H4D	108.9	C28X—C29X—H29D	91.5
C3B—C4B—H4D	108.9	H29C—C29X—H29D	105.2
H4C—C4B—H4D	107.7	C29X—C30X—C31X	110.9 (9)
O2B—C5B—C6B	121.3 (2)	C29X—C30X—H30C	119.6
O2B—C5B—C4B	120.9 (2)	C31X—C30X—H30C	111.3
C6B—C5B—C4B	117.8 (2)	C29X—C30X—H30D	103.1
C1B—C6B—C5B	119.1 (2)	C31X—C30X—H30D	105.6
C1B—C6B—C7B	121.9 (2)	H30C—C30X—H30D	104.9
C5B—C6B—C7B	118.9 (2)	C32X—C31X—C30X	108.7 (9)
C6B—C7B—C8B	108.6 (2)	C32X—C31X—H31C	104.8
C6B—C7B—C14B	110.29 (19)	C30X—C31X—H31C	116.8
C8B—C7B—C14B	113.1 (2)	C32X—C31X—H31D	102.5
C6B—C7B—H7B	108.2	C30X—C31X—H31D	114.1
C8B—C7B—H7B	108.2	H31C—C31X—H31D	108.6
C14B—C7B—H7B	108.2	C33X—C32X—C31X	110.3 (10)
C13B—C8B—C9B	118.5 (2)	C33X—C32X—H32C	112.9
C13B—C8B—C7B	122.2 (2)	C31X—C32X—H32C	106.1
C9B—C8B—C7B	119.3 (2)	C33X—C32X—H32D	113.4
O3B—C9B—C8B	121.3 (3)	C31X—C32X—H32D	104.1
O3B—C9B—C10B	121.8 (3)	H32C—C32X—H32D	109.4
C8B—C9B—C10B	116.8 (2)	C32X—C33X—H33D	106.2
C9B—C10B—C11B	113.5 (2)	C32X—C33X—H33E	111.4
C9B—C10B—H10C	108.9	H33D—C33X—H33E	109.5
C11B—C10B—H10C	108.9	C32X—C33X—H33F	110.8
C9B—C10B—H10D	108.9	H33D—C33X—H33F	109.5
C11B—C10B—H10D	108.9	H33E—C33X—H33F	109.5
			
C13A—O1A—C1A—C6A	10.7 (3)	O1B—C1B—C2B—C3B	155.9 (2)
C13A—O1A—C1A—C2A	−167.9 (2)	C1B—C2B—C3B—C20B	−70.1 (3)
C6A—C1A—C2A—C3A	20.0 (4)	C1B—C2B—C3B—C21B	168.7 (2)
O1A—C1A—C2A—C3A	−161.5 (2)	C1B—C2B—C3B—C4B	50.2 (3)
C1A—C2A—C3A—C4A	−49.6 (3)	C20B—C3B—C4B—C5B	64.7 (3)
C1A—C2A—C3A—C20A	70.9 (3)	C21B—C3B—C4B—C5B	−174.7 (2)
C1A—C2A—C3A—C21A	−168.2 (2)	C2B—C3B—C4B—C5B	−55.7 (3)
C20A—C3A—C4A—C5A	−64.5 (3)	C3B—C4B—C5B—O2B	−147.8 (2)
C21A—C3A—C4A—C5A	175.4 (2)	C3B—C4B—C5B—C6B	32.9 (3)
C2A—C3A—C4A—C5A	56.4 (3)	O1B—C1B—C6B—C5B	178.5 (2)
C3A—C4A—C5A—O2A	153.4 (3)	C2B—C1B—C6B—C5B	−1.8 (4)
C3A—C4A—C5A—C6A	−31.2 (3)	O1B—C1B—C6B—C7B	−6.4 (4)
O1A—C1A—C6A—C5A	−170.5 (2)	C2B—C1B—C6B—C7B	173.3 (2)
C2A—C1A—C6A—C5A	7.8 (4)	O2B—C5B—C6B—C1B	177.9 (2)
O1A—C1A—C6A—C7A	7.6 (4)	C4B—C5B—C6B—C1B	−2.7 (3)
C2A—C1A—C6A—C7A	−174.0 (2)	O2B—C5B—C6B—C7B	2.7 (3)
O2A—C5A—C6A—C1A	173.2 (2)	C4B—C5B—C6B—C7B	−178.0 (2)
C4A—C5A—C6A—C1A	−2.2 (3)	C1B—C6B—C7B—C8B	18.8 (3)
O2A—C5A—C6A—C7A	−5.0 (4)	C5B—C6B—C7B—C8B	−166.2 (2)
C4A—C5A—C6A—C7A	179.6 (2)	C1B—C6B—C7B—C14B	−105.7 (3)
C1A—C6A—C7A—C8A	−22.5 (3)	C5B—C6B—C7B—C14B	69.3 (3)
C5A—C6A—C7A—C8A	155.6 (2)	C6B—C7B—C8B—C13B	−19.1 (3)
C1A—C6A—C7A—C14A	102.3 (3)	C14B—C7B—C8B—C13B	103.7 (3)
C5A—C6A—C7A—C14A	−79.6 (3)	C6B—C7B—C8B—C9B	160.0 (2)
C6A—C7A—C8A—C13A	22.2 (3)	C14B—C7B—C8B—C9B	−77.2 (3)
C14A—C7A—C8A—C13A	−102.2 (3)	C13B—C8B—C9B—O3B	−178.8 (3)
C6A—C7A—C8A—C9A	−156.9 (2)	C7B—C8B—C9B—O3B	2.0 (4)
C14A—C7A—C8A—C9A	78.7 (3)	C13B—C8B—C9B—C10B	3.9 (4)
C13A—C8A—C9A—O3A	177.5 (2)	C7B—C8B—C9B—C10B	−175.2 (2)
C7A—C8A—C9A—O3A	−3.3 (4)	O3B—C9B—C10B—C11B	144.8 (3)
C13A—C8A—C9A—C10A	−5.0 (4)	C8B—C9B—C10B—C11B	−37.9 (4)
C7A—C8A—C9A—C10A	174.2 (2)	C9B—C10B—C11B—C23B	176.2 (3)
O3A—C9A—C10A—C11A	−145.0 (3)	C9B—C10B—C11B—C22B	−63.2 (3)
C8A—C9A—C10A—C11A	37.5 (3)	C9B—C10B—C11B—C12B	57.0 (3)
C9A—C10A—C11A—C22A	64.4 (3)	C10B—C11B—C12B—C13B	−44.3 (3)
C9A—C10A—C11A—C12A	−56.3 (3)	C23B—C11B—C12B—C13B	−164.1 (3)
C9A—C10A—C11A—C23A	−174.6 (3)	C22B—C11B—C12B—C13B	76.2 (3)
C22A—C11A—C12A—C13A	−75.9 (3)	C9B—C8B—C13B—O1B	−172.2 (2)
C23A—C11A—C12A—C13A	163.5 (3)	C7B—C8B—C13B—O1B	6.9 (4)
C10A—C11A—C12A—C13A	44.7 (3)	C9B—C8B—C13B—C12B	8.4 (4)
C9A—C8A—C13A—O1A	171.9 (2)	C7B—C8B—C13B—C12B	−172.5 (2)
C7A—C8A—C13A—O1A	−7.2 (4)	C1B—O1B—C13B—C8B	8.1 (3)
C9A—C8A—C13A—C12A	−6.3 (4)	C1B—O1B—C13B—C12B	−172.4 (2)
C7A—C8A—C13A—C12A	174.5 (2)	C11B—C12B—C13B—C8B	13.5 (4)
C1A—O1A—C13A—C8A	−10.8 (3)	C11B—C12B—C13B—O1B	−166.0 (2)
C1A—O1A—C13A—C12A	167.7 (2)	C6B—C7B—C14B—C19B	60.2 (3)
C11A—C12A—C13A—C8A	−15.4 (4)	C8B—C7B—C14B—C19B	−61.6 (3)
C11A—C12A—C13A—O1A	166.1 (2)	C6B—C7B—C14B—C15B	−113.7 (2)
C6A—C7A—C14A—C19A	−58.8 (3)	C8B—C7B—C14B—C15B	124.5 (2)
C8A—C7A—C14A—C19A	63.0 (3)	C19B—C14B—C15B—O4B	−177.5 (2)
C6A—C7A—C14A—C15A	123.0 (2)	C7B—C14B—C15B—O4B	−3.5 (4)
C8A—C7A—C14A—C15A	−115.2 (3)	C19B—C14B—C15B—C16B	3.2 (3)
C19A—C14A—C15A—O4A	−170.3 (2)	C7B—C14B—C15B—C16B	177.2 (2)
C7A—C14A—C15A—O4A	7.9 (4)	O4B—C15B—C16B—C17B	147.8 (2)
C19A—C14A—C15A—C16A	8.6 (4)	C14B—C15B—C16B—C17B	−32.9 (3)
C7A—C14A—C15A—C16A	−173.2 (2)	C15B—C16B—C17B—C18B	55.7 (3)
O4A—C15A—C16A—C17A	−162.9 (2)	C15B—C16B—C17B—C24B	−65.6 (3)
C14A—C15A—C16A—C17A	18.1 (3)	C15B—C16B—C17B—C25B	174.6 (2)
C15A—C16A—C17A—C25A	−166.8 (2)	C24B—C17B—C18B—C19B	69.2 (3)
C15A—C16A—C17A—C18A	−46.9 (3)	C25B—C17B—C18B—C19B	−170.8 (2)
C15A—C16A—C17A—C24A	73.0 (3)	C16B—C17B—C18B—C19B	−51.4 (3)
C25A—C17A—C18A—C19A	172.6 (2)	C15B—C14B—C19B—O5B	−179.2 (2)
C16A—C17A—C18A—C19A	52.2 (3)	C7B—C14B—C19B—O5B	6.9 (4)
C24A—C17A—C18A—C19A	−68.2 (3)	C15B—C14B—C19B—C18B	0.6 (4)
C15A—C14A—C19A—O5A	176.3 (2)	C7B—C14B—C19B—C18B	−173.3 (2)
C7A—C14A—C19A—O5A	−1.9 (4)	C17B—C18B—C19B—O5B	−154.9 (2)
C15A—C14A—C19A—C18A	−2.6 (4)	C17B—C18B—C19B—C14B	25.3 (4)
C7A—C14A—C19A—C18A	179.2 (2)	C28—C29—C30—C31	169 (2)
C17A—C18A—C19A—O5A	151.4 (2)	C29—C30—C31—C32	178 (2)
C17A—C18A—C19A—C14A	−29.7 (3)	C30—C31—C32—C33	175 (4)
C13B—O1B—C1B—C6B	−8.4 (3)	C28X—C29X—C30X—C31X	171 (3)
C13B—O1B—C1B—C2B	171.9 (2)	C29X—C30X—C31X—C32X	−179 (3)
C6B—C1B—C2B—C3B	−23.8 (3)	C30X—C31X—C32X—C33X	177 (3)

### Hydrogen-bond geometry (Å, °)

**Table:**

*D*—H···*A*	*D*—H	H···*A*	*D*···*A*	*D*—H···*A*
O5B—H5OB···O5A	0.82	1.69	2.488 (2)	163
O1W—H2W1···O4A^i^	0.89	2.03	2.918 (3)	175
N1—H1N1···O4A^ii^	0.88	1.94	2.755 (3)	155
N1—H2N1···O1W	0.87	2.01	2.860 (3)	167
C4B—H4C···O2A^i^	0.97	2.48	3.353 (4)	150
C12A—H12B···O3A^iii^	0.97	2.41	3.261 (3)	146
C12B—H12C···O3B^iv^	0.97	2.36	3.238 (3)	150
C18A—H18A···O5B	0.97	2.55	3.227 (3)	126
C26—H26C···O4B	0.96	2.42	3.244 (4)	144
C27—H27B···O3B	0.96	2.34	3.055 (4)	131

Symmetry codes: (i) −*x*, −*y*+1, *z*+1/2; (ii) −*x*+1/2, *y*+1/2, *z*+1/2; (iii) *x*+1/2, −*y*+1/2, *z*; (iv) *x*−1/2, −*y*+3/2, *z*.
